# Does Ocean Sunfish *Mola* spp. (Tetraodontiformes: Molidae) Represent a Risk for Tetrodotoxin Poisoning in the Portuguese Coast?

**DOI:** 10.3390/md20100594

**Published:** 2022-09-23

**Authors:** Miguel Baptista, Ana Catarina Braga, Rui Rosa, Pedro Reis Costa

**Affiliations:** 1 MARE—Marine and Environmental Sciences Centre/ARNET—Aquatic Research Network, Laboratório Marítimo da Guia, Faculdade de Ciências, Universidade de Lisboa, 2750-005 Lisbon, Portugal; 2 IPMA—Portuguese Institute for the Ocean and Atmosphere, Av. Alfredo Magalhães Ramalho, n° 6, 1495-165 Lisbon, Portugal; 3 S2AQUA—Collaborative Laboratory, Association for a Sustainable and Smart Aquaculture, Av. Parque Natural da Ria Formosa s/n, 8700-194 Olhão, Portugal; 4 Departamento de Biologia Animal, Faculdade de Ciências, Universidade de Lisboa, 1349-063 Lisboa, Portugal; 5 CCMAR—Centre of Marine Sciences, Campus de Gambelas, University of Algarve, 8005-139 Faro, Portugal

**Keywords:** tetrodotoxin, poisonous fish, seafood safety, emerging toxins

## Abstract

Tetrodotoxin (TTX) is a potent neurotoxin naturally occurring in terrestrial and marine organisms such as pufferfish. Due to the risk of TTX poisoning, fish of Tetraodontidae family and other puffer-related species must not be placed in the EU markets. This restriction applies to fish of the family Molidae even though no data on toxins’ occurrence is available. In this study, the presence of TTX and its analogues was investigated in the main edible tissue (the white muscle) and the main xenobiotics storage organ (the liver) of ocean sunfish *Mola* spp. (*n* = 13) from the South Portuguese coast. HILIC-MS/MS analyses did not reveal TTX in the analyzed samples, suggesting an inexistent or very limited risk of TTX poisoning.

## 1. Introduction

Tetrodotoxin (TTX) is a potent neurotoxin responsible for foodborne illnesses associated with consumption of certain fish species of the Tetraodontidae family, such as those belonging to the group of pufferfishes. In many countries of Eastern Asia, such as Japan, China and Korea, the consumption of pufferfish is a delicacy that often leads to human poisoning if proper evisceration and separation of the toxic fish tissues is not carried out before meal preparation [1,2]. Consumption of pufferfish in Europe is reduced or inexistent. Still, food safety authorities established that species and fishery products derived from the Tetraodontidae fish family cannot be placed on the EU market. Since no sufficient data on fish toxicity or TTX occurrence in autochthonous fish was available, other potentially poisoning fish such as those phylogenetically related to Tetraodontidae, namely the Molidae, Diodontidae, and Canthigasteridae were also banned from entering the EU markets as a precautionary measure (EC 853/2004) [3].

TTX is now considered an emerging toxin that may represent a risk to EU seafood consumers. The rise in awareness came after a human poisoning due to the ingestion, not of fish or fish products, but of a large marine gastropod. High TTX levels in the whole soft body of a trumpet shell *Charonia lampas* led to acute intoxication and hospitalization of a person in Spain, in 2007 [4]. After this incident, attempts have been made to investigate the presence of TTX and its analogues in seafood species, with only significant values being found again in *C. lampas* from the Portuguese coast [5,6,7]. Nevertheless, it was the presence of TTX in bivalve molluscs from Greece, the UK and the Netherlands [8,9,10,11] that led the European Food Safety Authority (EFSA) to state a scientific opinion on the risks for public health related to the TTX [12]. According to the EFSA scientific panel of experts, a concentration lower than 44 µg TTX equiv. kg^−1^ of shellfish meat is not expected to result in adverse effects in humans. But, to the best of our knowledge, Japan is the only country that has set a regulatory limit. The limit was based on mouse bioassays and is equivalent to 2 mg TTX kg^−1^ [13,14]. Also according to the EFSA, the most appropriate method for TTX determination is based on liquid chromatography with tandem mass spectrometry detection using hydrophilic interaction chromatography (HILIC-MS/MS).

In response to the EFSA scientific opinion on TTX, several research studies were carried out to investigate the presence of this toxin in seafood species from the EU waters. The presence of TTX and their seasonal variability was then assessed in bivalve molluscs from Portugal, Spain, France and Italy [15,16,17,18]. Although some results suggested a potential risk for consumers due to TTX concentration exceeding the EFSA safety limit, the very most of the bivalve samples analysed only revealed trace levels [15,16]. These results contrast with the high TTX values determined in native Guinean puffer *Sphoeroides marmoratus* from Madeira Island [19] or the invasive *Lagocephalus sceleratus*, a Lessepsian migrant that has been causing huge environmental and social issues in the Mediterranean Sea [20,21].

Both *Sphoeroides marmoratus* and *Lagocephalus sceleratus* are fish species of the Tetraodontidae family, and therefore must not be placed in the EU markets, especially due to their confirmed accumulation of extremely high levels of TTX. However, not all Tetraodontidae fish species accumulate TTX in their tissues, and thus do not represent a risk for human consumption, as is the case of the oceanic puffer *Lagocephalus lagocephalus* [19,20]. The same may apply to other fish species, not only of the Tetraodontidae family but puffer-related species of the Molidae, Diodontidae and Canthigasteridae families, which were never or only poorly tested for TTX.

During the late 1990′s and early 2000′s, the ocean sunfish *Mola* spp. were a fishery target reaching captures of 12 and 13 tons in Portugal and Ireland, respectively [22]. With the EU Regulation EC 853/2004 entering in force in 2004, landings of this fish species were interdicted. However, there is no evidence of ocean sunfish accumulating or vectoring TTX and its analogues. To the best of our knowledge, only two studies investigated the presence of TTX in ocean sunfish [23,24]. The earliest study performed a TTX screening in several puffer-related fish species, including 3 specimens of ocean sunfish (presumably *Mola mola*) captured in Japan, in 1987. The 3 sunfish specimens tested negative [23]. Later, attempts were made to clarify the toxicity of causative sharptail mola (*Masturus lamceolatus*) concerning with a food poisoning in Taiwan [24]. TTX was not detected by means of LC-MS in the remained uncooked sunfish fillets, neither in other sunfish (*Ranzania laevis*) obtained in the Taiwanese markets [24].

The present study aims to investigate by HILIC-MS/MS the presence of TTX and its analogues in the edible and non-edible tissues of the ocean sunfish *Mola* spp. from the Portuguese southern coast. To the best of our knowledge this is the first study investigating the presence of TTX in Molidae fish species in Europe.

## 2. Results and Discussion

**Figure 1:**
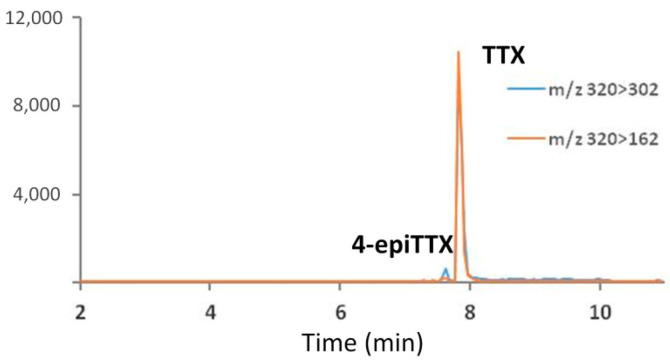
Multiple reaction monitoring (MRM) chromatogram of tetrodotoxin (TTX) obtained by HILIC-MS/MS analysis in spiked (25 ng mL^−1^) muscle extract of sunfish *Mola* spp.

TTX and its analogues were not detected in either the muscle (main edible portion) or liver of any ocean sunfish analysed (*n* = 13). The results are in agreement with the study carried out by [23] who analysed the liver of three presumably *Mola mola* specimens caught from Manazuru, Kanawaga, Japan, in 1987. Analyses carried out by [23] via mouse bioassay, HPLC, UV spectrophotometry and GC-MS did not indicate the presence of TTX or its analogues. Similarly, in the present study, using a recently updated HILIC-MS/MS method, no TTX was found in the tissue samples of the 13 specimens caught in the South Portuguese coast. The LOQ (10× S/N) on TTX spiked tissue samples was 0.8 ng TTX mL^−1^, corresponding to 16 µg TTX kg^−1^. Figure 1 shows the chromatogram of an extract of muscle tissue spiked with 25 ng TTX mL^−1^. The method recovery was 91.7 ± 4%.

According to the results shown in the present study, there is no indication that ocean sunfish accumulate TTX, and consequently the risk for human consumption may be reduced or inexistent. However, this study was limited to 13 individuals captured in spring, which, together with the autumn is one of the two seasons of the year when significant peaks of sunfish abundance occur in the South Portuguese coast [25]. These abundances were found to be correlated with an increase in both water temperature and productivity, which are the general conditions favoring TTX accumulation in marine organisms [9]. Moreover, ocean sunfish diet, in particular of smaller individuals as those included in the present study, consists of coastal benthic and pelagic species, such as bivalve mollusks, gastropods, crustaceans and small fish species [26] that may potentially act as TTX vectors [5,6,7,8,19].

While no TTX was found in ocean sunfish from the Portuguese coast, trace elements that may be toxic at low concentrations, such as cadmium and lead, were determined in white muscle and liver of such fish from the Portuguese coast [27]. The greatest elemental content was found in the liver, where higher metal pollution index was registered, highlighting this organ as an important storage organ for contaminants. The liver can be used as a metal contamination indicator, a role that may also be applied for the occurrence of marine toxins. In the present study, the absence of TTX in the liver highly suggests that sunfish are not exposed and do not harbor this toxin. Therefore, assuming the phylogenetic relationship of ocean sunfish and pufferfish to establish the risk of TTX poisoning appears to not be accurate. The lack of evidence of ocean sunfish as a TTX-bearing organism suggests that fisheries restrictions due to risk of TTX poisoning may have been be incorrectly defined. It is important, however, to highlight that the conservation status of ocean sunfish is uncertain and some degree of protection may be required. In that case fisheries restrictions must be issued by the competent authorities on fisheries management and wildlife conservation. In addition to demystify sunfish as a poisonous fish, this study highlights the need to further investigate the ecology, taxonomy and species distribution.

## 3. Materials and Methods

### 3.1. Samples Collection

**Figure 2:**
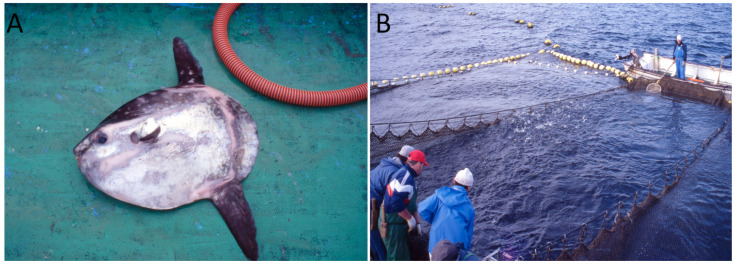
Ocean sunfish *Mola* sp. (**A**) captured in set-net targeting tuna (**B**), off Olhão, south coast of Portugal.

Very recent genetic and morphological studies have shed light on the phylogeny of ocean sunfishes—Molidae [28,29,30]. One important revelation is that, contrary to past belief, *Mola mola* is not the sole *Mola* species inhabiting the North Atlantic Ocean, co-existing with *M. alexandrini*. Accordingly, as it is possible that both *M. mola* and *M. alexandrini* specimens were analysed in this study, we adopt the terms ocean sunfish and/or *Mola* spp. when referring to the examined specimens and their counterparts in the wild. Regardless, as such species distinction is still far from being acknowledged by society in general, including fishermen, for the purpose of this work, no species distinction is actually required. Thirteen ocean sunfish (*Mola* spp.) were captured in April 2016 (spring) in the South Portuguese coast, where high TTX levels have been determined in large marine snail gastropods, *Charonia lampas* [7]. The sunfish were collected at a set-net targeting tuna (Tunipex), off Olhão (Figure 2). The specimens varied between 38.4 and 94.2 cm in total length, and their weight ranged from 3.4 to 49.7 kg. Six were female and other 6 were male. The sex was undetermined for the smaller specimen.

Two tissues/organs were dissected for TTX analysis, namely the white muscle (edible) and liver (contaminants storage organ). Samples were storage frozen until toxins extraction.

### 3.2. Toxin Extraction and Analysis

#### 3.2.1. Reagents

All reagents used for toxins extraction and analysis were of analytical grade or higher. Acetic acid glacial (100%, p.a.), methanol (>99.8%, p.a.), LC–MS additive grade ammonium hydroxide solution (NH_4_OH, 25% as NH_3_), formic acid (98–100%) and acetonitrile (analytical grade) were obtained from Sigma-Aldrich (Sintra, Portugal); ammonium formate (>99% purity) was from Fluka and hydrochloric acid (37%) from Panreac. Water was purified using a Milli-Q 185 Plus system from Millipore. The toxin standard solution for TTX (>99% purity) was sourced from CIFGA (Lugo, Spain).

#### 3.2.2. Toxin Extraction

A portion of white muscle, the main edible tissue, and liver, the main storage organ where high levels of xenobiotics are usually accumulated, were dissected from each animal. The fish tissues were homogenized in a blender and 5 g taken into a 30-mL test tube. Toxins were extracted with 5 mL of 1% acetic acid by vortexing for 90 s and heating for 5 min in a boiling water bath. Samples were cooled down until room temperature was achieved and were again vortexed for another 90 s. After that, centrifugation of the samples for 10 min at 3000× *g* was conducted. A clean-up step using Graphitised Carbon SPE was carried out following the method described by [9,31]. Then, 5 µL of 25% *v*/*v* of NH_3_ was added to 1 mL of the supernatant and was centrifuged at 1000× *g* for 1 min before performing the SPE clean-up step. The ENVI-Carb cartridge (Supelclean, Supelco, Sigma-Aldrich, Sintra, Portugal) was conditioned with 3 mL of 20% MeCN + 1% *v*/*v* HOAc and 3 mL of 0.025% *v*/*v* NH_3_. A 400 µL aliquot of sample extract was loaded onto the cartridge and washed with 700 µL deionized water. Toxins elution and collection were carried out through the addition of 2 mL 20% MeCN + 0.25% acetic acid. The eluted extract was mixed and then diluted by transferring 100 µL to a vial and adding 300 µL of acetonitrile before analysis.

#### 3.2.3. TTX Analysis by HILIC-MS/MS

The LC-MS/MS equipment consisted of an Agilent 1290 Infinity coupled to a triple quadrupole mass spectrometer, an Agilent 6470. The chromatographic separation was conducted with a hydrophilic interaction liquid chromatography (HILIC) UHPLC column (1.7 µm, 2.1 mm × 150 mm Waters Acquity Glycan BEH Amide column) in conjunction with a Waters VanGuard BEH Amide guard column (Waters, Lisbon, Portugal). The chromatographic separation was performed using the conditions described in the Standard Operating Procedure (SOP) for determination of TTX provided by the European Union Reference Laboratory [32]. Elution was achieved using a binary eluent system: Eluent A water/formic acid/NH_4_OH (500:0.075:0.3 *v*/*v*/*v*) and eluent B acetonitrile/water/formic acid (700:300:0.1 *v*/*v*/*v*). A gradient started at 98% B at 0.4 mL min^−1^ for the first 5 min, 98–50% B for the next 2.5 min, and this composition was kept for 1.5 min but the flow rate was linearly increased to 0.5 mL min^−1^ until 9.0 min, and then B reverted to 98% by 9.5 min, flow rate ramped to 0.8 mL min^−1^ at 10.0 min, held until 10.6 min, and dropped back to 0.4 mL min^−1^ until 11.0 min. Two multiple-reaction-monitoring (MRM) transitions from the protonated ions were monitored for TTX and TTX derivatives as described in: For TTX and 4-epiTTX the transitions monitored were the *m*/*z* 320.1 > 302.1 and *m*/*z* 320.1 > 162.1, for 11-deoxyTTX *m*/*z* 304.1 > 286.1 and *m*/*z* 304.1 > 162.1, for 4,9-anhydroTTX *m*/*z* 302.1 > 284.1 and *m*/*z* 302.1 > 162.1, and *m*/*z* 290.1 > 272.1 and *m*/*z* 290.1 > 162.1 for 11-norTTX-6(R/S)-ol. The optimized source settings were as follows: Gas temperature 150 °C, gas flow 13 L min^−1^, nebulizer 50 psi, sheath gas temperature 400 °C, sheath gas flow 12 L min^−1^ and capillary voltage 2500 V. Linearity of the calibration curves was validated for TTX standards prepared in solvent and white muscle sunfish matrix. A five-point calibration curve of TTX with a correlation >0.995 was set up for quantification.

## 4. Conclusions

This is the first study investigating the presence of TTX and its analogues in ocean sunfish *Mola* spp. from European waters. In Europe this species is considered a poisonous fish and fishery products derived from this fish family are prohibited on the market. According to our analyses, no TTX was detected in the ocean sunfish captured in the South Portuguese coast, suggesting that phylogenetic relationship between ocean sunfish and pufferfish should not be used to establish the risk of TTX poisoning. Further studies, including higher number of specimens and other tissues, such as the gonads and skin, should be conducted to confirm these findings and improve knowledge on sunfish ecology. Nevertheless, the present study is of key importance to demystify sunfish as poisonous fish.
